# Organization and Complexity of the Yak (Bos Grunniens) Immunoglobulin Loci

**DOI:** 10.3389/fimmu.2022.876509

**Published:** 2022-05-09

**Authors:** Mingli Wu, Haidong Zhao, Xiaoqin Tang, Wanxia Zhao, Xiaohua Yi, Qi Li, Xiuzhu Sun

**Affiliations:** ^1^ College of Animal Science and Technology, Northwest A&F University, Yangling, China; ^2^ College of Grassland Agriculture, Northwest A&F University, Yangling, China

**Keywords:** yak, IgH, Igλ, Igκ, structure of the genomic, diversity of expression

## Abstract

As important livestock in Qinghai-Tibet Plateau, yak provides meat and other necessities for Tibetans living. Plateau yak has resistance to diseases and stress, yet is nearly unknown in the structure and expression mechanism of yak immunoglobulin loci. Based on the published immunoglobulin genes of bovids (cattle, sheep and goat), the genomic organization of the yak immunoglobulin heavy chain (IgH) and immunoglobulin light chain (IgL) were described. The assemblage diversity of IgH, Igλ and Igκ in yak was similar to that in bovids, and contributes little to the antibody lineage compared with that in humans and mice. Somatic hypermutation (SHM) had a greater effect on immunoglobulin diversity in yak than in goat and sheep, and in addition to the complementarity-determining region (CDR), some loci in the framework region (FR) also showed high frequency mutations. CDR3 diversity showed that immunological lineages in yak were overwhelmingly generated through linkage diversity in IgH rearrangements. The emergence of new high-throughput sequencing technologies and the yak whole genome (2019) publication have greatly improved our understanding of the immune response in yaks. We had a more comprehensive analysis of yak immunoglobulin expression diversity by PE300, which avoided the disadvantage of missing low-frequency recombination in traditional Sanger sequencing. In summary, we described the schematic structure of the genomic organization of yak IgH loci and IgL loci. The analysis of immunoglobulin expression diversity showed that yak made up for the deficiency of V(D)J recombinant diversity by junctional diversity and CDR3 diversity. In addition, yak, like cattle, also had the same ultra-long IgH CDR3 (CDR3H), which provided more contribution to the diverse expression of yak immunoglobulin. These findings might provide a theoretical basis for disease resistance breeding and vaccine development in yak.

## 1 Introduction

Humoral immunity is an immune mechanism by which B lymphocytes produce antibodies to achieve the purpose of protection. The key process of humoral immunity is the production of highly effective and short-lived effector B lymphocytes, which secrete antibodies to remove antigens. B lymphocytes, also called “B cells” could generate nearly an infinite number of antibodies with diverse binding sites through combinatorial rearranging of large numbers of duplicated genes that encode antibody specificity, by modifying the joining sites using exonucleases and terminal deoxynucleotidyl transferase (TdT) and then targeting these rearrangements for SHM using nucleases like activation-induced cytidine deaminase (AID) (1). There was a specific B cell receptor (BCR) on the surface of each B cell, which directly binds the antigen to humoral immunity. When an antigen invades the body, only B cells with complementary BCR can combine with the antigen, then receive selective stimulation, and then clone on a large scale (clonal selection theory) (2). Classical immunoglobulin has a Y-shaped structure which consists of two identical IgL and two identical IgH (3). The variable region of IgH and Igκ/Igλ constitute the epitopes of immunoglobulin, which directly determines the specificity and affinity of the antibody to antigen by binding with antigen. The constant region mainly regulates immune cells and effector molecules or cells. The physicochemical and structural properties of CDRs between Igκ and Igλ were quite different, but there was no significant difference in their pairing with IgH (4).

In response to a variety of pathogenic microorganisms in the environment, the body has evolved a variety of mechanisms, such as V(D)J recombination, gene conversion (GCV), SHM and class switch recombination (CSR), resulting in immunoglobulin diversity (5). In addition to CSR targeting constant regions, the other four mechanisms enrich the types of antibodies by increasing the diversity of variable regions. Recombination-activating genes 1/2 (RAG 1/2) and high-mobility group (HMG) protein initiate V(D)J recombination, specifically recognize recombination signal sequence (RSS) on the flank of the recombinant gene and form RAG complex, and then cut the RSS, repair the DNA double strand break (DSB) and complete rearrangement (6–9).

The schematic structure of the genomic organization and diversity of immunoglobulin in some ruminants had been reported. In 2016, Dr. Ma completed the genome organization structure of bovine IgH. The IgH consists of 43 VH genes, 23 DH genes and 12 JH genes. The bovine IgH had an ultra-long CDR3H not found in other species, encoding up to 65 amino acids (AA) (10, 11). Ultra-long CDR3H had previously been found in IgNAR of sharks and was thought to exist only in cartilaginous fish. But then longer ultra-long CDR3H was found in cattle, which presents a unique ‘stalk and knob’ structure in the crystal of ultra-long CDR3H antibodies. Different paired combinations of cysteine (Cys) in CDR3H form disulfide bonds, thus forming a variety of different spatial configurations of antigen binding sites under the same CDR3H sequence (12, 13). Goat IgH contains eight VH genes, three DH genes and six JH genes, and only three VH genes and three DH genes and two JH genes were involved in the VDJ recombination. The composition of IgH was similar between sheep and goats, but the number of germ line genes is much less than that of cattle. In bovids, due to the low level of κ gene expression in cattle, there were few studies on the κ chain in cattle, Dr. Qin and Dr. Yu completed the study on the structure and expression diversity of immunoglobulin κ gene in sheep and goat in 2015 and 2020, respectively (14, 15). The sheep λ chain consists of 32 Vλ genes, three Jλ genes and two λ genes, and only seven Vλ genes have potential functions. The goat λ chain consists of 35 Vλ genes, three Jλ genes and three λ genes, just like sheep, there are only seven Vλ genes with potential function. The number of V, D and J genes germline genes in cattle was slightly higher than that in sheep and goats, but far lower than that in humans and mice.

Known as the boat of the plateau, yak live on the Qinghai-Tibet plateau at an altitude of 3 km above sea level and showed satisfactory cold and disease resistance. Therefore, the study of the structure and diversity mechanism of the yak immunoglobulin gene could be of academic and practical significance. Although there was a breakthrough in the structure of immunoglobulin in cattle and sheep, the insufficient basic knowledge of yak immune function hindered our ability to breed and maintain optimal yak health. Therefore, the study of the immunoglobulin gene map and diversity mechanism of yak is of great significance for disease resistance breeding, disease prevention and vaccine production of yak.

## 2 Materials and Methods

All experimental procedures were carried out accordance according to the Regulations on the Administration of Affairs Concerning Experimental Animals approved by the State Council of the People’s Republic of China. The research was approved by the Institutional Animal Care and Use Committee of Northwest A&F University.

### 2.1 Analysis of the yak VH, Vλ and Vκ Segments

The yak genome (Bos grunniens, assembly BosGru3.0) was provided by NCBI (https://www.ncbi.nlm.nih.gov/genome/?term=yak). Immunoglobulin V gene was searched in yak genome with NCBI BLAST (Basic Local Alignment Search Tool) using the immunoglobulin V genes of cattle, goat, sheep, human and mouse as templates (16). The same method was used to obtain the immunoglobulin constant region genes in yak. FUZZNUC (http://embossgui. sourceforge.net/demo/fuzznuc.html) was used to find RSS to locate the D genes and J genes. V gene domains (FRs or CDRs) and classifications (potential functional gene, open reading frame (ORF), and pseudo gene) were divided according to IMGT (https://www.imgt.org/) (17).

### 2.2 Sequence Computations

DNA sequence editing, alignments and comparisons were performed using the SnapGene (18).

### 2.3 Phylogenetic Analysis

The phylogenetic tree was constructed using Mega version 7.0 (www.mega.com/) (19). ClustalW was used to compare multiple DNA sequences of the constructed tree. Each VH, Vλ and Vκ subgroup was randomly selected as a sequence among functional genes. The VH, Vλ and Vκ sequences used in this study (excluding yak) were as follows: human (Homo sapiens): VH1, L22582; VH2, X62111; VH3, X92206; VH4, Z12367; VH5, M99686; VH6, X92224; VH7, L10057; Vκ1, V01577; Vκ2, X63403; Vκ3, X12686; Vκ4, Z00023; Vκ5, X02485; Vκ6, X63399; Vκ7, X12682; Vλ1, Z73654; Vλ2, X97462; Vλ3, X97464; Vλ4, X57828; Vλ5, Z73672; Vλ6, Z73673; Vλ7, X14614; Vλ8, Z73650; Vλ9, Z73675; Vλ10, Z73676; Vλ11, D86996 (4, 20, 21). Mouse (Mus musculus): VH1, M34982; VH2, J00502; VH3, K01569; VH4, AC079273; VH5, X00163; VH6, AC073590; VH7, X03253; VH8, AC079181; VH9, L14368; VH10, AF064444; VH11, AC073563; VH12, AC073590; VH13, X55935; VH14, X55934; VH15, AC090843; VH16, AC073563; Vκ1, AJ231198; Vκ2, J00562; Vκ3, Y15968; Vκ4, AJ231209; Vκ5, AJ235974; Vκ6, AJ235962; Vκ7, AF044198; Vκ8, AJ235948; Vκ9, V00804; Vκ10, AF029261; Vκ11, AJ231256; Vκ12, AJ235951; Vκ13, AJ231273; Vκ14, AJ231241; Vκ15, AJ231269; Vκ16, AJ235936; Vκ17, AJ231258; Vκ18, AJ235966; Vκ19, AJ235935; Vλ1, J00590; Vλ3, M34597; Vλ4, M94349 (22, 23). Sheep (Ovis aries): VH1, H6; VH2, H7; VH3, H203; VH4, H16; VH5, H37; VH6, H168; VH7, H242; VH8, H17; VH9, H23 is from Charlton’s article; Vκ1, AF038133; Vκ2, AF038134; Vκ3, AF038137; Vκ4, AF038138; Vλ1, AF040908; Vλ2, AF040921; Vλ3, AF040914; Vλ4, AF040907; Vλ5, AF040906; Vλ6, AF038145 (14, 15, 24). Horse (Equus caballus): VH1, DQ125413; VH2, HM175886; VH3, HM176050; Vκ1, HM176113; Vκ9, HM176157; Vκ10, Vκ12, Vκ13, Vκ15, Vκ19, CM000391; Vλ1, KR190470; Vλ2, KR190471; Vλ3, KR190472; Vλ4, KR190473; Vλ8, KR190477; Vλ10, KR190479; Vλ14, KR190483; Vλ25, KR190494; Vλ81, KR190550; Vλ77, KR190546; Vλ20, KR190489 (25, 26). Cattle (Bos taurus): immunoglobulin rearranged VH, KT723008; Vλ1, HM596891; Vλ2, U32258; Vλ3, HM596897 (11, 27–29). Chinese alligator (Alligator sinensis): VH1-8, JQ479335 (30). Green anole (Anolis carolinensis): Vκ1, EU419646; Vκ2, EU419647; Vκ3, EU419651; Vκ24, EU419668; Vκ7, EU419655 (31). Chicken (Gallus domesticus): VH1, AB233003; Vλ1, AB061561 (32, 33). African clawed frog (Xenopus laevis): VH1, Y00380; VH2, M30025; VH3, M24675; VH4, M24676; VH5, M24677; VH6, M24678; VH7, M24679; VH8, M24680; VH9, M24681; Vκ1, L15570; type III V1, L76575; type III V2, L76577; type III V3, L76586; type III V4, L76579; type III V5, L76576 (34–36). zebrafish (Danio rerio): VH1, BX649502 (37). Common carp (Cyprinus carpio): Cpi1, AB091112; Cpi2, AB091113; Cpe1, AB073328; Cpf1, AB073332; Cpg1, AB073335; Cpc1, AB035728; Cpb4, AB035730 (38, 39). Nurse shark (Ginglymostoma cirratum): VH1, M92851; NS3, EF114759; NS4, GU109471; NS5, AY720853 (7, 40).

### 2.4 Animals, RNA Isolations

In this study, three healthy and unrelated yaks (3 - 4 years old) were selected. RNA from spleens was extracted using the traditional Trizol method (TaKaRa, Dalian) (41).

### 2.5 Cloning of the Expressed Yak IgH, Igλ and Igκ Chain Genes by 5’ Race PCR and Sequencing

The SMARTer RACE 5’/3’ Kit (Takara, Dalian) was used to amplify the IgH, Igλ and Igκ of yak. The specific primers for IgH chain were as follows: yak-IgH-F, 5’-AAGCAGTGGTATCAACGCAGAGT-3’; yak-IgH-R, 5’- AGCTCACGCAGGACACCAG-3’. The specific primers for Igκ chain were as follows: yak-Igκ-F, 5’- AAGCAGTGGTATCAACGCAGAGT-3’; yak-Igκ-R, 5’- AGATGGATGGCTGAGCATCA-3’. The specific primers for Igλ chain were as follows: yak-Igλ-F, 5’-AAGCAGTGGTATCAACGCAGAGT-3’; yak-Igλ-R, 5’-GTGACCGAGGGTGCGGACTT-3’. Since the length of DH genes were random, and PE300 sequencing requires that the fragment length should not exceed 550 bp, IgH was sequenced by sanger sequencing, and Igλ and Igκ were sequenced by PE300. The IgH PCR product was cloned into pMD-19T vector (TaKaRa, Dalian) and the sequence was determined by sanger sequencing. 100 clones of each sample were sequenced. The PCR products of Igλ and Igκ were directly sequenced by PE300.

### 2.6 V(D)J Combinatorial Diversity Analysis, SHM Statistics and CDR3 Calculations

All items were clustered with germline V genes, and each germline V gene was a cluster. The clone (from FR1 to FR3) that most matched the germ line fragment was analyzed by VH expression and base substitution, and the variation sites and frequencies were calculated by Mega version 7.0 and Excel software.

Editing and alignment of nucleotide sequences were described in Section 2.4. Mega version 7.0 was used to determine the types of V(D)J gene in all cloned fragments and calculated the combinatorial diversity of all clones. According to the grouping of V gene by Mega version 7.0, the base substitution of clone sequence (FR1-FR3) was analyzed by germline V gene as a template, the nucleotide variation frequencies and types of all sites were calculated. CDR3 region refers to the sequence between ‘YYC’ and ‘WGXG’ (‘FGXG’ in IgL) (42). ‘YYC’, ‘WGXG’ and ‘FGXG’ are relatively conservative, but with a little variation. It is necessary to truncate the CDR3 region of the cloned fragment according to the actual conditions. The random deletion sequence and random insertion sequence (non-templated (N) nucleotides and palindromic (P) nucleotides) were calculated according to the germline V, D and J genes.

High-throughput sequencing and Sanger sequencing were completed by Shanghai Biotech Company. The general process of high-throughput sequencing is as follows:

(1) Perform 5’ RACE according to experimental requirements to recover PCR products and send samples to the sequencing department; (2) PCR products of the samples were tested for quality, and the library was prepared after qualified: Illumina bridge PCR compatible primers were introduced, and the DNA fragments were purified; (3) In order to obtain uniform long cluster effect and high-quality sequencing data, Qubit3.0 fluorescence quantifier was used for library concentration determination and quality inspection. After passing the library, the library was sequenced on the computer, and PE300 double-terminal sequencing program was run to obtain 300 bp double-terminal sequencing reads and splice them. (4) Conduct bioinformation analysis after obtaining sequencing data spliced from the company: Reads were screened, and those without amplified primers (F: AAGCAGTGGTATCAACGCAGAGT, yak-IgH-R: AGCTCACGCAGGACACCAG, yak-Igκ-R: AGATGGATGGCTGAGCATCA, yak-Igλ-R: GTGACCGAGGGTGCGGACTT), and with sequencing length less than 400 bp were removed. After obtaining coarse screening data, the sequencing sequences were analyzed as follows; IMGT was used to compare the sequencing data with the reference V gene, D gene and J gene, and the sequences of the V gene and J gene could be compared at the same time for subsequent analysis; MEGA (version7.0) was used to divide all sequences into V gene, (D gene), J gene and junction fragment; The neighbor-joining tree was constructed by MEGA (version7.0) to identify the template V gene, D gene and J gene corresponding to all sequences. V gene is downloaded into Excel format after ClustaIW alignment for SHM calculation; Blast (version2.2.30) was used to find the alignment information of V, D and J sequences, and output N and P nucleotides to generate specific information; (5) Statistical classification of immunoglobulin expression spectrum data: The number of sequences using different V, (D) and J clans in each sample was counted without removing repeated reads, and the results were converted to the percentage of the number of sequences using each V clan in the total number of sequencing; CDR3 sequence of each read was counted, the length of each CDR3 fragment was calculated, and the CDR3 length distribution data of each sample was analyzed;The number of bases in each reads nucleotide and the distribution of N and P nucleotide in each individual were calculated; The SHM frequency was counted. The formula was used in this study: SHM frequency= the number of observed mutant bases/the total number of sequenced bases; SHM analysis, classify observable mutant bases, count the number of mutations from A, T, C and G to the other three bases, and convert the results into the percentage of the number of each mutation in observable mutant bases; (6) GraphPad Prism 6 was used for mapping and statistical analysis.

## 3 Results

### 3.1 Structure of the Genomic Organization and Phylogenetic Analysis of Yak IgH

**Figure 1:**
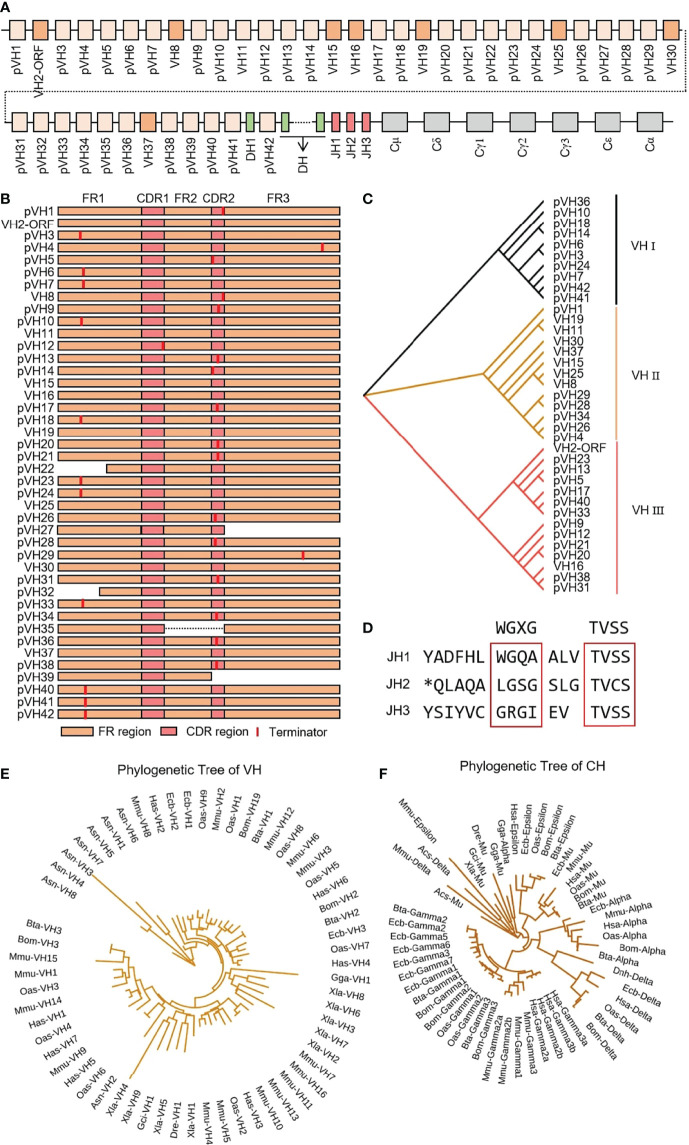
Schematic structure of the genomic organization of Yak IgH and its relatedness with representative vertebrate species. **(A)** Schematic structure of the genomic organization of Yak IgH; **(B)** The structural variation of Yak VH; **(C)** The phylogenetic tree of yak VH; **(D)** The structural variation of Yak JH, *: termination codon **(E)** The phylogenetic tree of VH in yak and representative vertebrate species; **(F)** The phylogenetic tree of different CH gene family between yak and representative vertebrate species.

IgH spans about 475 kb on chromosome 17 in yak, including 42 VH genes, 55 DH genes, three JH genes, one μ gene, one δ gene, three γ genes, one ϵ gene and one α gene (**Figure 1A**). Only eight VH genes had potential functions, including leader exons, complete translation initiation codons, splice fragments and downstream RSSs, but did not include stop codon or frameshift mutations. VH2 gene was ORF because the 104th AA is not conserved Cys. Five VH genes structures were incomplete, and the remaining 27 VH genes were terminated prematurely (**Figure 1B**). VH genes were divided into three clans, and the potentially functional VH genes were mainly concentrated in clan II (**Figure 1C**). All three JH genes had no features of ‘WGXG’ or ‘TVSS’ (**Figure 1D**). In addition, all 55 DH genes exist in at least one optional reading frame, indicating their potential capabilities. The homology of yak VH genes with human, mouse, cattle, sheep, horse, green anole lizard, chicken, xenopus laevis, zebrafish and nurse shark were analyzed by MrBayes 3.1 software. The tree was constructed from FR1-FR3 nucleotide sequence. **Figure 1E** shows that the evolution of the three VH clans of yak is most closely related to the three VH clans of cattle, followed by the clan I, clan III and clan VII of sheep. The evolutionary relationship of yak constant region gene was similar to VH gene, and yak constant region genes had the highest similarity to cattle, followed by yak (**Figure 1F**).

### 3.2 Diversity of IgH

#### 3.2.1 VDJ junctions in the expressed IgH chains

**Figure 2:**
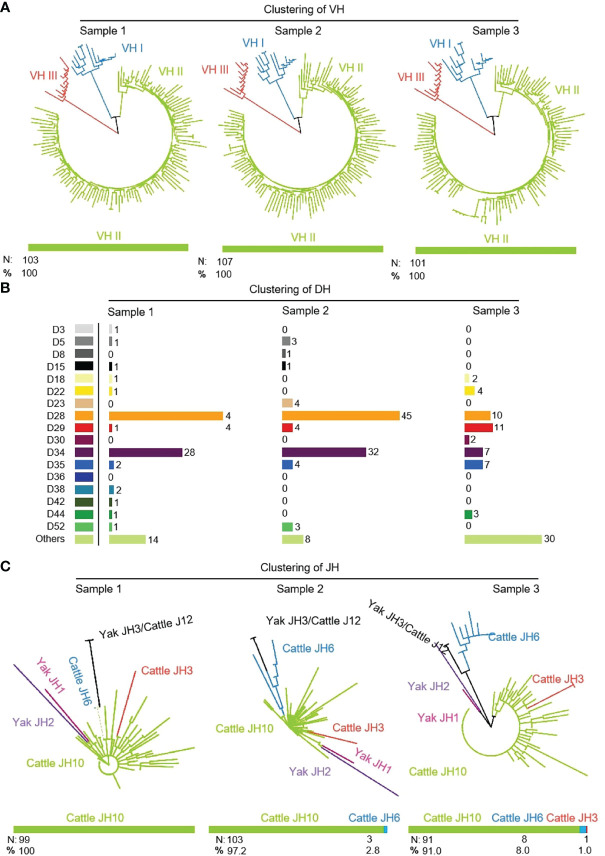
The VH, DH and JH genes usage in Yak IgH. **(A)** Expression of the yak VH; **(B)** Expression of the yak DH; **(C)** Expression of the yak JH.

311 (n1 = 103, n2 = 107, n3 = 101) IgH sequences were cloned from three yak spleen samples. The VH, DH and JH genes of the rearranged sequence were grouped by germline genes with potential function through Mega version 7.0. Because there is no potential functional JH gene found in the yak genome, bovine JH genes were used as germline JH genes. Yak had the same preferences for VH, DH and JH, and all three individuals only use the VH clan II (**Figure 2A**). Due to the high similarity of the VH genes in VH clan II and the existence of SHM in VH gene, it is impossible to accurately determine which germline VH all clones belong to. The DH gene mainly expresses DH28 and DH34 (**Figure 2B**). JH gene was not expressed in three yak germline JH genes, among which the highest expression frequency of the bovine JH10 gene was 100%, 97.2%, 91%. Three clones expressed the bovine JH6 gene in individual 2, six clones expressed the bovine JH6 gene and one in individual 3. The bovine JH3 gene was expressed in six clones (**Figure 2C**).

#### 3.2.2 CDR3H diversity

**Figure 3:**
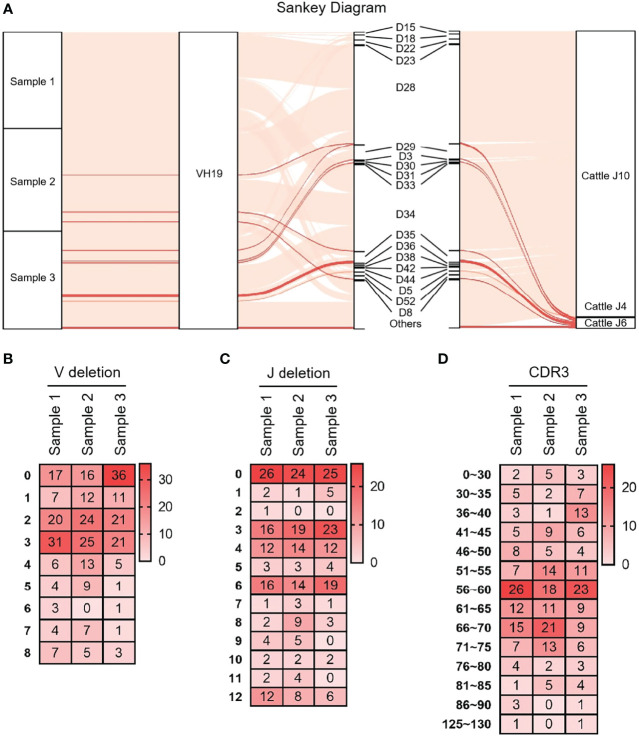
The diversity of VDJ recombination and CDR3 length in yak IgH. **(A)** The sankey diagram of VDJ recombination in yak IgH; **(B)** the random deletion nucleotides of VH gene in yak IgH; **(C)** the random deletion nucleotides of JH gene in yak IgH; **(D)** the nucleotides of CDR3 in yak IgH.

As shown in **Figure 3A** that the cloned sequences were aligned by germline VHs, DHs and JHs. There were few combinations of VDJ expressed, and most clones express the combination of ‘VH clan II-DH28/34- bovine JH10’. It showed that the diversity of yak IgH combinations is single. All clones were grouped and aligned manually. Some sequences were randomly deleted at the 3’ end of V gene and the 5’ end of J gene. As shown in **Figures 3B, C**, the length of the deleted fragment at the 3’ end of V gene was mainly 0 bp, 2 bp and 3 bp. The length of the 5’ end deletion fragment of the JH gene was mainly concentrated on 0 bp, 3 bp and 6 bp. The sequence between the conserved AA ‘YYC’ of the VH gene and the conserved AA ‘WGXG’ of the JH gene was called the CDR3H region, including the 3’ end of VH gene, complete DH gene and the 5’ end of JH gene. The CDR3H length of yak ranges from 5 to 43 AA (with an average of 19.28 AA) (**Figure 3D**).

#### 3.2.3 SHM analysis of VH (FR1-FR3)

**Figure 4:**
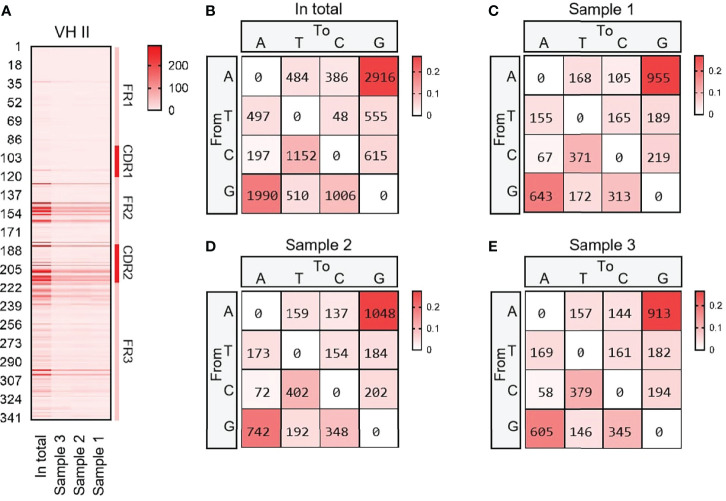
The somatic hypermutation (SHM) of yak IgH. **(A)** The distribution of SHM in yak IgH; **(B–E)** The base mutation types of SHM in yak IgH.

VH15 was used as a template for SHM analysis with all cloned sequences. The variation frequency of each point was shown in **Figure 4A**. The variation frequency of FR1 was the lowest among the five regions, and the high frequency variation region was concentrated in the CDR1-CDR2 region. Several high-frequency variation sites were found at the 3’ end of FR3. According to the statistics of single base variation types of all loci, the base substitution between A and G was significantly higher than other base substitution forms (**Figure 4B–E**), accounting for 7.1%, 10.2% and 7.3% of the variation frequency of the three individuals respectively, which was consistent with the SHM model of cattle and sheep.

### 3.3 Structure of the Genomic Organization and Phylogenetic Analysis of Yak Igκ

**Figure 5:**
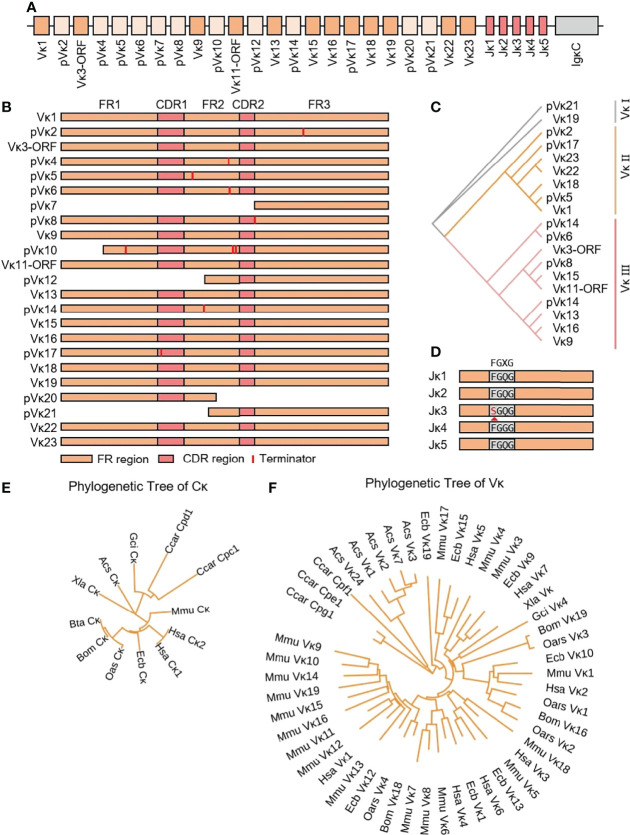
Schematic structure of the genomic organization of Yak IgL(κ) and its relatedness with representative vertebrate species. **(A)**: Schematic structure of the genomic organization of Yak IgL(κ); **(B)** The structural variation of Yak Vκ; **(C)** The phylogenetic tree of yak Vκ; **(D)** The structural variation of Yak Jκ; **(E)** The phylogenetic tree of κ in yak and representative vertebrate species; **(F)** The phylogenetic tree of different Vκ gene family between yak and representative vertebrate species.

The yak Igκ was located on chromosome 9 (GenBank: CM016698) with full-length 175 kb (**Figure 5A**). Yak Igκ contained 23 Vκ genes, of which nine had potential functions, the 23rd AA of both ORFs was not conserved Cys, five had large fragment deletions and the rest of the fragments were terminated prematurely (**Figure 5B**). According to the sequence similarity, all Vκs could be divided into three clans, which were similar to sheep Vκs (**Figure 5C**). Five Jκ genes followed Vκs, which Jκ3 didn’t contain a conserved ‘FGXG’ motif (**Figure 5D**). κ was located downstream of Jκ, with a conserved 23 bp nucleotide interval on the 5’ end. MrBayes 3.1 software was used to analyze the clan of Vκ gene. The tree was constructed from the nucleotide sequence FR1-FR3. k gene of yak was most closely related to cattle, followed by sheep and horse, which had the same relationship with IgH (**Figure 5E**). As shown in **Figure 5F**, yak had the highest homology with cattle. The Vκ I of yak was clustered with Vκ3 of sheep, Vκ2 gene clan of yak was clustered with Vκ4 of sheep, and Vκ3 gene clan of yak was clustered with Vκ2 of sheep. The κ gene of yak had the highest homology with cattle, followed by sheep and horses (**Figure 5F**).

### 3.4 Diversity of Igκ

#### 3.4.1 V-J junctions in the expressed Igκ chain

**Figure 6:**
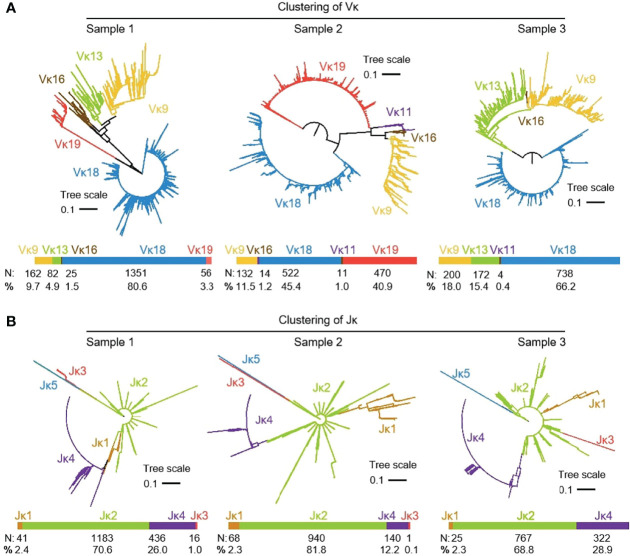
The Vκ and Jκ genes usage in yak IgL (κ). **(A) **Expression of the yak Vκ; **(B)** Expression of the yak Jκ.

3939 (n1 = 1676, n2 = 1149 and n3 = 1114) recombinant Igκ sequences were cloned from three yak spleen samples. Six kinds of Vκ were expressed in all clones, namely Vκ9, Vκ11, Vκ13, Vκ16, Vκ 18 and Vκ 19, respectively. The expression rate of Vκ11 was 1.22% and 0.4% in individuals 2 and 3, respectively. The highest expression of Vκ18 was 80.6%, 45.4% and 66.2%, respectively (**Figure 6A**). The results of the Jκ cluster showed that Jκ2 was mainly used in three individuals, and the utilization rates were above 68%. The frequency of Jκ4 expression was second only to Jκ2. It was found that Jκ1 and Jκ3 were used in a few clones. The Jκ distribution of the three yaks was similar (**Figure 6B**).

#### 3.4.2 CDR3κ diversity

**Figure 7:**
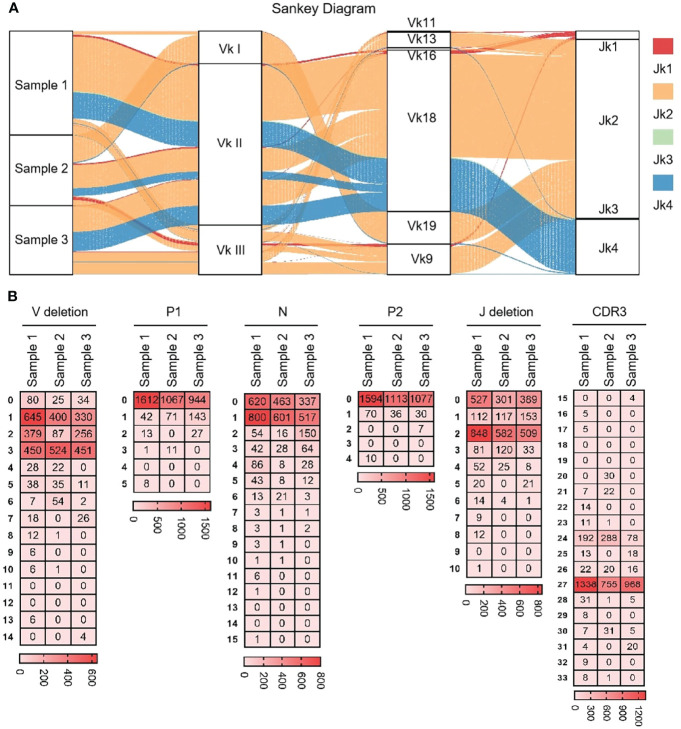
The diversity of VJ recombination and CDR3 length in yak IgL(κ). **(A)** The sankey diagram of VJ recombination in yak IgL(κ); **(B)** the nucleotides distribution of V deletion, P1 nucleotide, N nucleotide, P2 nucleotide, J deletion and CDR3 in yak IgL(κ).

As shown in **Figure 7A** that the cloned sequences were aligned by germline Vκs and Jκs. There were few recombinat sequences of expressed Vκ-Jκ, and the combination ‘Vκ18-Jκ2’ was expressed in most clones. These results showed that Igκ was biased in the process of recombination, but its diversity was higher than that of IgH. All clones were manually grouped and compared, and random deletions of the 3’ end of Vκ gene and the 5’ end of Jκ gene were counted. As shown in **Figure 7B**, the random deletion length of 3’ end of Vκ and the 5’ end of Jκ was mainly 1-3 bp. Random addition of non-template sequences between the Vκ gene and Jκ gene, including N nucleotide and P nucleotide. Statistics showed that the insertion of N and P nucleotides mainly focus on 0 - 1 bp. The sequence between the conserved AA ‘YYC’ of Vκ and the conserved AA ‘FGXG’ of Jκ was called CDR3κ region, which included the 3’ end of Vκ, the 5’ end of Jκ and the random insertion sequence between Vκ and Jκ. The length of CDR3κ in yak was mainly 8 - 9 AA.

#### 3.4.3 SHM analysis in the expressed Vκ region

**Figure 8:**
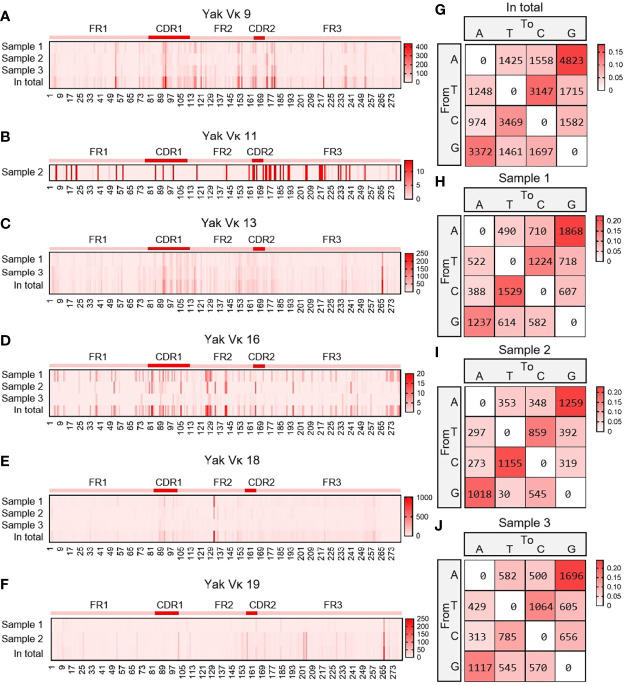
The somatic hypermutation (SHM) of yak IgL(κ). **(A–F)** The distribution of SHM in yak IgL(κ); **(G–J)** The base mutation types of SHM in yak IgL(κ).

As shown in **Figures 8A–F**, six Vκ genes were expressed, namely Vκ9, Vκ11, Vk13, Vκ16, Vκ18 and Vκ19. All clones were grouped into six Vκ genes, and SHM analyzed were performed. The change frequency of each spot is shown in **Figures 8A–F**. The high-frequency changes were not completely concentrated in the CDRs, and high-frequency mutations also appeared at some sites in the FR2 region. According to the statistics of the types of base substitution at all sites, the frequency of base exchange between ‘A’ and ‘G’ was the highest, followed by that between ‘C’ and ‘T’ (**Figures 8G–J**). This study found that the SHM pattern of yak was the same as that of humans, mice and sheep, which indicated that SHM was evolutionarily conservative.

**Figure 9:**
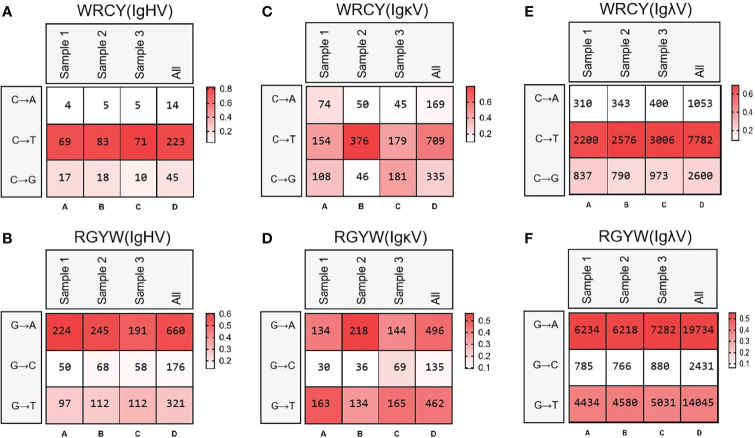
Hot spot mutation frequency of SHM in IgH, Igκ, Igλ. **(A)** IgH SHM in “WRCY”; **(B)** IgH SHM in “RGYW”; **(C)** Igκ SHM in “WRCY”; **(D)** Igκ SHM in “RGYW”; **(E)** Ig**λ** SHM in “WRCY”; **(F)** Ig**λ** SHM in “RGYW”.

Then, the frequency of C/G mutation in AID hotspot WRCY/DGYW was analyzed, that is, the frequency of C/G mutation in the hotspot and the frequency of the total mutant base. The mutation frequencies of IgH, Igκ and Igλ were highest when C mutated to T and G mutated to A (**Figure 9**).

### 3.5 Structure of the Genomic Organization and Phylogenetic Analysis of Yak Igλ

**Figure 10:**
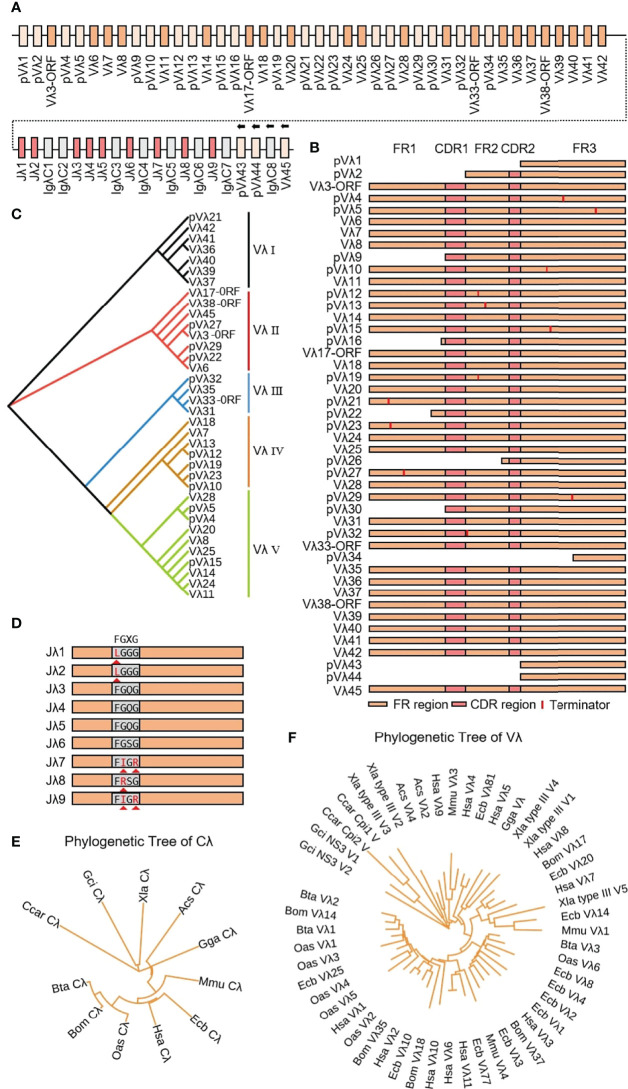
Schematic structure of the genomic organization of Yak IgL(λ) and its relatedness with representative vertebrate species. **(A)** Schematic structure of the genomic organization of Yak IgL(λ); **(B)** The structural variation of Yak Vλ; **(C)** The phylogenetic tree of yak Vλ; **(D)** The structural variation of Yak Jλ; **(E)** The phylogenetic tree of Cλ in yak and representative vertebrate species; **(F)** The phylogenetic tree of different Vλ gene family between yak and representative vertebrate species.

Yak Igλ was located on chromosome 16 (GenBank: CM016705) with full-length 1287 kb, including 45 Vλ genes, nine Jλ genes and eight λ genes. The genes from pVλ1 to λ7 were closely distributed, only 346 kb, and the downstream genes are far away and the transcription direction is opposite to the chromosome direction (**Figure 10A**). Based on the classification rules of the IMGT database, 18 of the 45 Vλ genes had potential functions, four were ORFs, eight were structurally incomplete, and 15 were pseudo genes (**Figure 10B**). According to the homology of nucleic acid sequence, all Vλ genes could be divided into five clans. The Vλ genes with potential functions were mainly concentrated in clan I and clan II (**Figure 10C**). Nine Jλ genes appeared upstream of eight λ genes. Unlike human, mouse and cattle, the Jλ genes in yak did not exist in the form of (Jλ-λ)n pairing, but randomly distributed in the upstream of λ. Jλ1, Jλ2, J9λ7, J9λ8 and Jλ9 did not contain conservative ‘FGXG’ (**Figure 10D**). Phylogenetic analysis of Vλ genes and λ genes in yak was carried out by Bayesian method, and the serial numbers of related genes were shown in 2.3. Yak λ was grouped with cattle, sheep and horse λ (**Figure 10E**). The tree was constructed by the nucleotide sequences FR1-FR3. A gene with potential functions was randomly selected from each Vλ clan, and an evolutionary tree system was constructed. As shown in **Figure 10F**, a close relationship between yak and cattle, horse and sheep, was observed, and yak Vλ clan I clustered with horse Vλ3, yak Vλ clan II clustered with horse Vλ20, yak Vλ clan III clustered with sheep Vλ2, yak Vλ clan IV clustered with horse Vλ10, yak Vλ clan V clustered with cattle Vλ2.

### 3.6 Diversity of Igλ

#### 3.6.1 V-J junctions in the expressed Igλ chain

**Figure 11:**
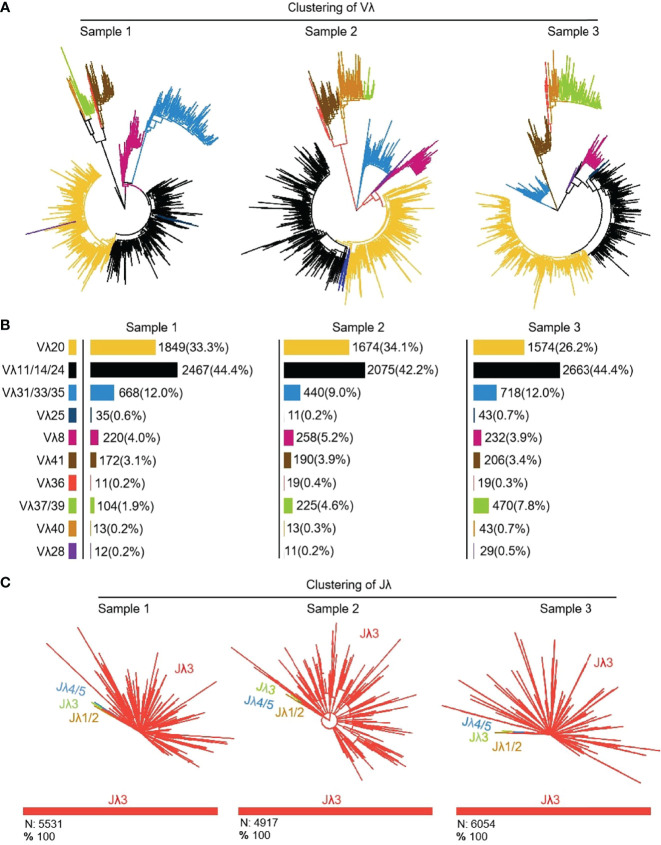
The Vλ and Jλ genes usage in yak IgL (λ). **(A, B)** Expression of the yak Vλ; **(C)** Expression of the yak Jλ.

16464 (n1 = 5551, n2 = 4916, n3 = 5997) Igλ recombinant sequences were cloned from 3 yak spleen samples. All clones expressed 10 kinds of Vλ, namely Vλ8, Vλ11, Vλ20, Vλ25, Vλ28, Vλ31, Vλ36, Vλ37, Vλ40 and Vλ41 respectively (**Figures 11A, B**). Among the three individuals, the expression of Vλ11 (yellow) was the highest, followed by Vλ20 (black). Vλ11 and Vλ20 belong to the clan V, and among the eight kinds of Vλ genes with low expression, except for Vλ33 (blue, clan III), the expression level was less than 10%. All Vλ genes were expressed in similar proportion in the three individuals. Jλ3 was expressed by all clones (**Figure 11C**).

#### 3.6.2 CDR3λ diversity

**Figure 12:**
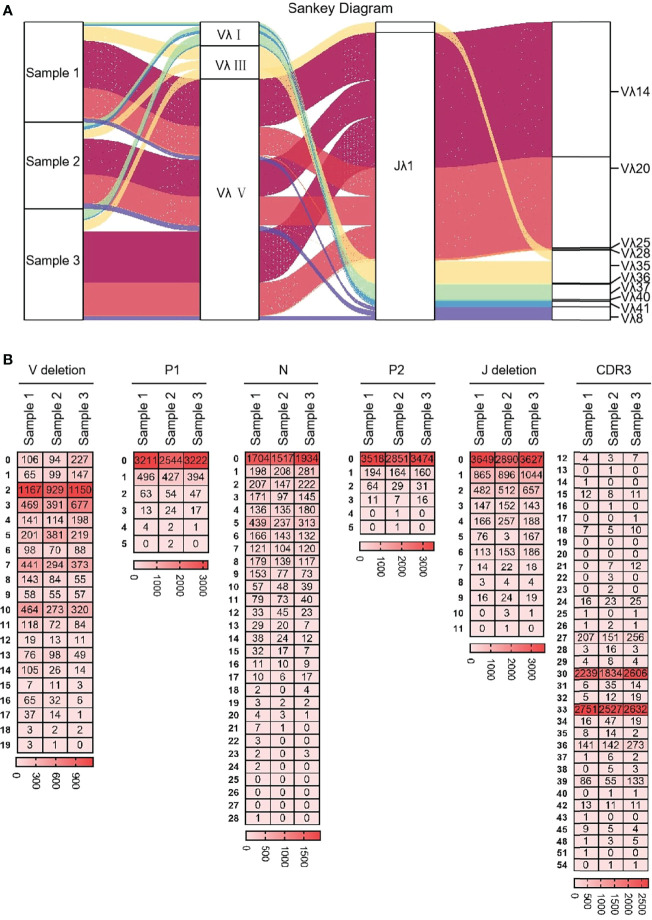
The diversity of VJ recombination and CDR3 length in yak IgL(λ). **(A)** The sankey diagram of VJ recombination in yak IgL(λ); **(B)** the nucleotides distribution of V deletion, P1 nucleotide, N nucleotide, P2 nucleotide, J deletion and CDR3 in yak IgL(λ).

Ten Vλ genes were expressed and one Jλ was expressed, so there were 10 types of Vλ-Jλ recombination types in the clones, with the highest recombination probability of Vλ14-Jλ1 (**Figure 12A**). The recombination probability of Vλ clan V was the highest, followed by Vλ clan III and Vλ clan I. All clones were manually grouped and compared, and the random deletions of Vλ 3’ end and Jλ 5’ end were counted. As shown in **Figure 12B**, the random deletion lengths of the 3’ end of Vλ and the 5’ end of Jλ were mainly 0 - 3 bp. The non-template sequences between Vλ and Jλ were randomly added, including N nucleotides and P nucleotides. The statistical results showed that the insertions of N and P nucleotide were mainly concentrated in 0 - 2 bp. The sequence between the conserved AA ‘YYC’ of Vλ and the conserved AA ‘FGXG’ of Jλ was called CDR3λ region, which includes the 3’ end of Vλ, the 5’ end of Jλ and the random insertion sequence between Vλ and Jλ. The length of yak CDR3λ was mainly 10 - 11 AA, which was longer than that of CDR3κ (**Figure 12B**).

#### 3.6.3 SHM analysis in the expressed Vλ region

**Figure 13:**
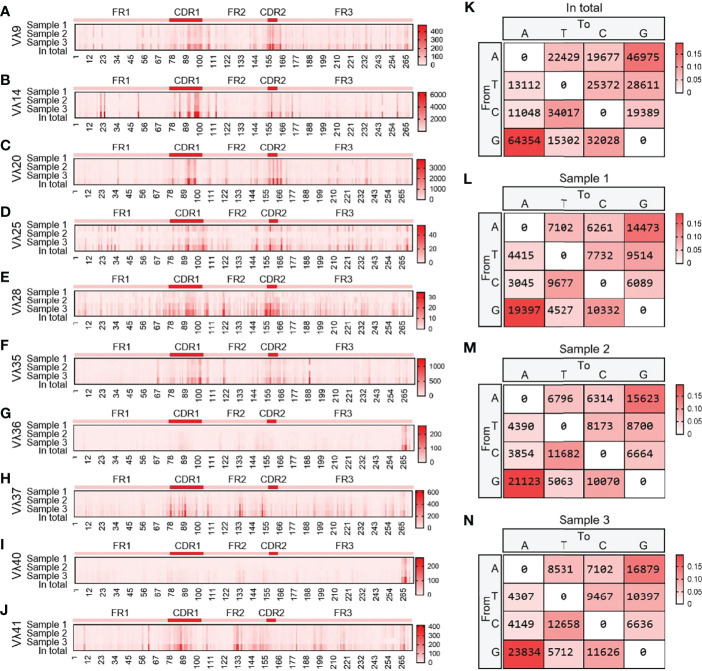
The somatic hypermutation (SHM) of yak IgL(λ). **(A–J)** The distribution of SHM in yak IgL(λ); **(K–N)** The base mutation types of SHM in yak IgL(λ).

10 Vλ genes were expressed, and all clones were grouped according to 10 Vλ genes, which were analyzed by SHM. The change frequency of each point is shown in **Figures 13A–J**. The high-frequency changes were not completely concentrated in the CDRs, and high-frequency mutations also appeared in FRs. According to the statistics of the base substitution types of all sites, the base exchange frequency between A and G was the highest (**Figures 13K–N**). In this study, it was found that the SHM pattern of yak Igλ was the same as that of IgH and Igκ.

## 4 Discussion

It is critical to understand the natural immune mechanisms that fight infection and how vaccination, biosafety, nutrition, livestock and management practices could be used to maintain and enhance immune protection (43). The sequence and structure of IgH constant genes in yak were similar to those in bovids (11). Interestingly, two μ genes with high similarity were found in the genome of cattle, but only one μ gene had been found in the genome of other bovids (yak, sheep and goat) (11, 24, 44, 45). Three JH genes were found in the yak genome, but none of them were transcribed. The three JH genes did not have typical ‘WGXG’ and ‘TVSS’ AA, which might be pseudogenes (45). The results showed that more than 95% of recombinant sequences in yak expressed bovine JH10 gene and 4% of recombinant sequences expressed bovine JH6 gene. By comparing JH gene sequences of yak and cattle, it was found that yak JH1 gene was highly similar to bovine JH1 gene, yak JH2 gene was highly similar to bovine JH5 gene, and yak JH3 gene was similar to bovine JH12 gene. Therefore, we speculated that the sequence between bovine JH5-JH6-μ1-…-JH7- JH8- JH9- JH10- JH11 might be missing in yak genome. The IgH span of yak was 475 kb, that of cattle was 678 kb, and that of JH5-JH6-μ1-…-JH7-JH8-JH9-JH10-JH11 was about 200 kb, which was consistent with the difference between the length of cattle and yak IgH. In the study of VDJ recombination of cattle, JH10 was recombined with the μ2 gene, and JH6 was recombined with μ1 gene. For these reasons, some JH genes and one μ gene might be missing in the yak genome. There was also a strong preference for yak DH genes in VDJ recombination. DH 28 and DH 34 were mainly used in VDJ recombination, which had obvious (YG)n AA characteristics, which was consistent with the characteristics of bovine DH8 gene (11). There were 42 germline VH genes in the yak genome, only 9 VH genes with potential function, all of which belong to clan II. The genes in clan II were about 90% homologous. Yak V gene contributed less to VDJ recombinant diversity, which was similar to horse. There were 54 species VH genes in horse, only VH4 and VH5 (clan II) were mainly used in VDJ recombination. **Figure 1E** showed that horse VH clan II and Yak VH clan II belong to the same branch (26). Since the VDJ recombination of bovine IgH was not reported. The three VH genes involved in VDJ recombination in goat belong to the clan I, but the VH genes used in goat were more abundant and less conservative than in other species, so the VH genes in goats contribute more to the diversity of VDJ recombination in bovids (24, 45). In summary, recombinant diversity of VDJ recombination in bovids contributes limited immunoglobulin diversity compared to human.

The previous researchers did not study the diversity of Igκ expression because the expression level of Igκ was extremely low. In this study, the Igκ of yak was amplified successfully, and the structure and diversity of Igκ were analyzed. The Igκ of yak had a typical structure of the genomic organization, containing only one κ gene, 5 Jκ genes and 23 Vκ genes. The κ genes were conserved in most mammals and were highly similar in sequence and number, but rabbits had two κ genes in particular. Yak, mouse, rabbit, horse and pig had five Jκ genes, while goat and sheep had only three Jκ genes and one was 19 bp irregular RSS (20, 25, 46, 47). Jκ1-4 was used in yak and horse VJ recombination and was similar in type and frequency of Jκ used (26). In this study, high-throughput sequencing found that six Vκ genes and four Jκ genes were used in VJ recombination, and Vκ16 and Jκ3 with extremely low frequency (less than 1%) were also found. The abundance of VJ recombination of Igκ in yak was higher than that in sheep and goats. Previous studies have found that Igλ of all species followed the rule of (Jλ-Cλ)n. The structure of the genomic organization of Igλ was Vλn-(Jλ-Cλ)_7_-Vλn in horse, Vλn-(Jλ-Cλ)_4_ in cattle, Vλn-(Cλ-Jλ)_3_-λ4 in pigs, Vn-(Jλ-Cλ)_2_, in sheep and Vλn- (Jλ-Cλ)_3_ in goat (15, 26). Interestingly, we found that yak Igλ did not follow such a rule, and the structure of the genomic organization of yak Igλ was Jλ1-Jλ2-λ1-λ2-Jλ3-Jλ4-Jλ5-λ3-Jλ6-λ4-Jλ7-λ5-Jλ8-λ6-Jλ9-λ7-λ8. 11 Vλ genes and four Jλ genes (Jλ1, Jλ4, Jλ6, Jλ7) were used in VJ recombination of Equine Igλ (25, 26). Cattle mainly expresses Vλ1a, Vλ1b, Jλ2 and Jλ3. Pig only Jλ2-λ2 and Jλ3-λ3 were used in recombination (47, 48). Only the combination of Jλ1-λ1 was found in the recombination sequence for sheep (14). 11 Vλ genes and four Jλ genes were used for VJ recombination of Equine Igλ (25, 26). The bovine Vλ1a, Vλ1b, Jλ2 and Jλ3 gene were mainly expressed. Jλ2-Cλ2 and Jλ3-Cλ3 have been used for recombination in pigs. Only Jλ1-Cλ1 combinations were found in the recombination sequence of sheep. Six Vλ genes and four Jλ genes were involved in the VJ recombination of yak (27, 29). The abundance of yak VJ recombination was close to that of horse but higher than that of goat, sheep and cattle.

Yak IgH, Igλ and Igκ showed a significant preference for J gene selection when V(D)J was recombined, and the IgH also had obvious preference for DH genes. Studies have shown that a single DH gene fragment could form its own CDR3H pedigree, which was sufficient to meet the needs of B cell development and immune function. However, multiple DH gene fragments are still necessary for full immune function. This study also found that the retention and development of CDR3H in specific species has its own characteristics in terms of length and AA utilization. CDR3H includes 3’ end of VH gene, DH gene, 5’ end of JH gene, V-D and D-J junction sequences (N nucleotides and P nucleotides). The random deletion of the 3’ end of V gene and the 5’ end of J gene, and the random insertion of N and P nucleotides were counted in this study (49). The random deletion of V gene mainly concentrated in 0 - 3 bp, with the largest deletion of 8 bp. The random deletion of J gene was mainly 0 bp, 3 bp, 4 bp and 6 bp, and the longest deletion was 12 bp. Compared with human (6.5 ± 1.7 bp), mouse (5.8 ± 1.7 bp) and cattle (8.8 ± 3.7 bp), the contribution of VH gene fragment to CDR3H length was smaller in yak (5.5 ± 2.1 bp) (11, 20, 50, 51). The contribution of Yak JH gene to CDR3H length was 7.6 ± 3.7 bp, and that of cattle was 15.1 ± 4.0 bp. The most notable and significant difference is the contribution length of DH to CDR3H. The DH contribution length of human and mouse was 14.3 ± 5.5 bp and 10.8 ± 4.7 bp, respectively. The contribution of bovine μ1 and μ2 to CDR3H length was 27.5 ± 8.5 bp and 43.1 ± 25.3 bp, respectively (52). The length of DH contribution of yak was 39.9 ± 13.7 bp, which was between bovine μ1 and μ2. Previous studies have found that cattle have an ultra-long CDR3H, and the longest CDR3H reaching 65 AA, which is not available in other species. By cloning yak IgH, we found the average length of CDR3H in yak was 58.0 ± 15.3 bp. Unfortunately, the longest CDR3 fragment did not match the DH expressed in the genome, which might be due to the missing of some fragment in genome splicing or the large variation of SHM in the CDR3H region. It is also possible that D-D fusion exists (53, 54). It was important since D-D fusions often result in long (at least 24 AA) and ultralong CDR3Hs (55). However, there was no evidence to prove whether D-D fusion really exists and the mechanism of D-D fusion, so D-D fusion was not further analyzed in this study (56). Previous studies had shown that ultra-long CDR3H was considered to be a unique and diverse form of bovine immunoglobulin production, but in this study, we confirmed that ultra-long CDR3H also exists in yak, and CDR3H includes V gene and was also realized by ultra-long DH. Dr. Ma found that the bovine DH gene of ultra-long CDR3H had a ‘YxYx’ feature, which also appeared in the ultra-long CDR3H sequence of yak (11). CDR3H was located in the center of the antigen binding site and plays a key role in determining the specificity and affinity of antibodies (43). DH gene was the main component of CDR3H and largely determines the length and AA composition of CDR3H. As the core element of VDJ recombination, DH gene seems to have different adaptations during the evolution of different animals. As the core element and contributor of CDR3H, DH genes showed large sequence variation among species and were less conserved than VH and JH genes. Thus, the DH gene in a particular species may be the key to producing more protective antibodies and fewer autoreactive antibodies in that species. The antibody of ultra-long CDR3H had a special structure and the ability to bind to special antigens. Additionally, ultra-long CDR3H resulted in “microfolds” allowing bovine IgH to bind antigens that would otherwise be inaccessible (12). The number of Yak VH, DH and JH genes was limited, and it was thought that IgH diversity was achieved through frequent recombination and endogenous mutations in the CDR3H region (57). It was still interesting to know exactly what advantages ultra-long CDRH3 confers on immunoglobulin function. For cattle and yaks, ultra-long CDR3H might have developed as another mechanism for maximizing diversification in the face of limited combinations of V(D)J. Alternatively, the ultra-long CDR3H might have evolved to optimize binding to antigens from rumen microbes or certain bovine specific pathogens which were not typically encountered by other vertebrates (12, 58, 59).

SHM was an important mechanism of antibody diversity after V(D)J recombination. The AA changes caused by the mutation could improve the affinity of antibodies (5, 60). The SHM of yak IgH had a preference, and the mutation frequency from A to G and G to A was the highest, followed by the mutation between A and T, which was the same as that of cattle, mice, zebrafish, etc., which indicates that the mutation pattern of SHM is evolutionarily conservative (11, 37). However, it is worth noting that in addition to CDR1 and CDR2, high-frequency SHM loci also exist on FR2, possibly because some germ line VH genes exist in unsplicing genome fragments, resulting in an incomplete template library. In addition, studies have shown that SHM is produced in order to increase the diversity of fetal bovine antibodies, and it has also been reported that the point mutations produced by SHM were mainly concentrated at 100-200 bp away from the gene transcription start site, which might be the reason why SHM was not concentrated in CDRs regions in yak (61, 62). The SHM pattern of the yak was different from sheep and goats. The average SHM of the VH gene, Vλ gene and Vκ gene was 8.26%, 6.61% and 16.75%. The SHM frequencies of two horses used in previous study were 8.35% and 7.79% for the VH, 10.24% and 10.27% for the Vλ, and 4.17% and 5.51% for the Vκ (26). The SHM frequencies of the yak VH gene were 7.1%, 10.2% and 7.3%, Vλ gene SHM frequencies were 6.92%, 8.26% and 7.58%, and Vκ gene SHM frequencies were 2.24%, 2.13% and 2.85%, respectively. The SHM frequency of yak H chain and λ chain was similar to that of other large livestock, but the SHM frequency of κ clan was much lower than that of other large livestock. SHM was an irreversible process, and the accumulated SHM were all favorable mutations, and SHM frequency was higher in species with more perfect germinal centers.

In this study, the diversity of yak IgH was analyzed by high-throughput sequencing - PE300, which greatly increased the number of reads analyzed (nλ=16464, nκ=3939), and some low-frequency recombinant sequences of VJ were found, such as Vλ25-Jλ3, Vλ28-Jλ3, Vλ36-Jλ3 and Vλ40-Jλ3. PE300 could avoid the omission of low-frequency expression genes due to the small number of samples. The differences between the findings of existing studies on Igκ in pigs might be influenced by the fact that too few items were sequenced. (47, 63). PE300 was not used for the IgH diversity study in this study, because yak had extremely long CDR3 (59, 64, 65).

By comparing the production mechanism of immunoglobulin diversity in different species, it was found that humans and mice had rich V(D)J recombination diversity, and they could produce rich immunoglobulin types through recombination to resist the invasion of different antigens (66). For species with less V(D)J recombination types, such as rabbit, it compensated for this limitation through GCV of different somatic cells and high levels of SHM, resulting in a more diverse antibody lineage than human or mouse. Structurally, rabbit antibodies rely more on IgL for antigen specificity, using longer CDR3L rings and interdomain disulfide bonds (67). In camels, B cells produce heavy chain only antibodies (HCAbs) in addition to regular antibodies with a IgH/IgL pairing configuration. These HCAbs contained some unusual structural features that further diversify the camel’s antibody library (68). In this study, by analyzing the structure and expression diversity of immunoglobulin gene in the yak, it was found that yak compensated for the restriction of little V(D)J recombination by ultra-long CDR3H and abundant SHM, which was consistent with the previous production mode of immunoglobulin diversity in cattle. However, the mechanism that relies on ultra-long CDR3H to increase immunoglobulin diversity had not been found in other species. Therefore, we hypothesized that the ultra-long CDR3H was the unique mechanism of the rich immunoglobulin diversity acquired during the evolution of the bovid. It was critical to understand the natural immune mechanisms that fight infection and how vaccination, biosafety, nutrition, livestock and management practices could be used to maintain and enhance immune protection. It was hoped that the study on yak immunoglobulin could provide basic theoretical knowledge for yak breeding and immunity research.

## Data Availability Statement

The datasets presented in this study can be found in online repositories. The names of the repository/repositories and accession number(s) can be found below: https://www.ncbi.nlm.nih.gov/, PRJNA805364.

## Ethics Statement

The animal study was reviewed and approved by Institutional Animal Care and Use Committee of Northwest A&F University.

## Author Contributions

HZ, MW, and XT analyzed the data; MW, XT, WZ, and QL wrote the manuscript; HZ, MW, WZ, QL, and XY carried out the experiment; XY and XS reviewed and edited the manuscript; HZ designed the experiment. All authors contributed to the interpretation of the results and writing of the manuscript. All authors contributed to the article and approved the submitted version.

## Funding

Project supported by National Natural Science Foundation of China (31972557).

## Conflict of Interest

The authors declare that the research was conducted in the absence of any commercial or financial relationships that could be construed as a potential conflict of interest.

## Publisher’s Note

All claims expressed in this article are solely those of the authors and do not necessarily represent those of their affiliated organizations, or those of the publisher, the editors and the reviewers. Any product that may be evaluated in this article, or claim that may be made by its manufacturer, is not guaranteed or endorsed by the publisher.

## Supplementary Material

Supplement 1 DEGs list
