# Supplementary figures and images for: Organization and Complexity of the Yak (Bos Grunniens) Immunoglobulin Loci
Source: Front Immunol. 2022 May 9;13:876509. doi: 10.3389/fimmu.2022.876509 (PMC9124968; doi:10.3389/fimmu.2022.876509)

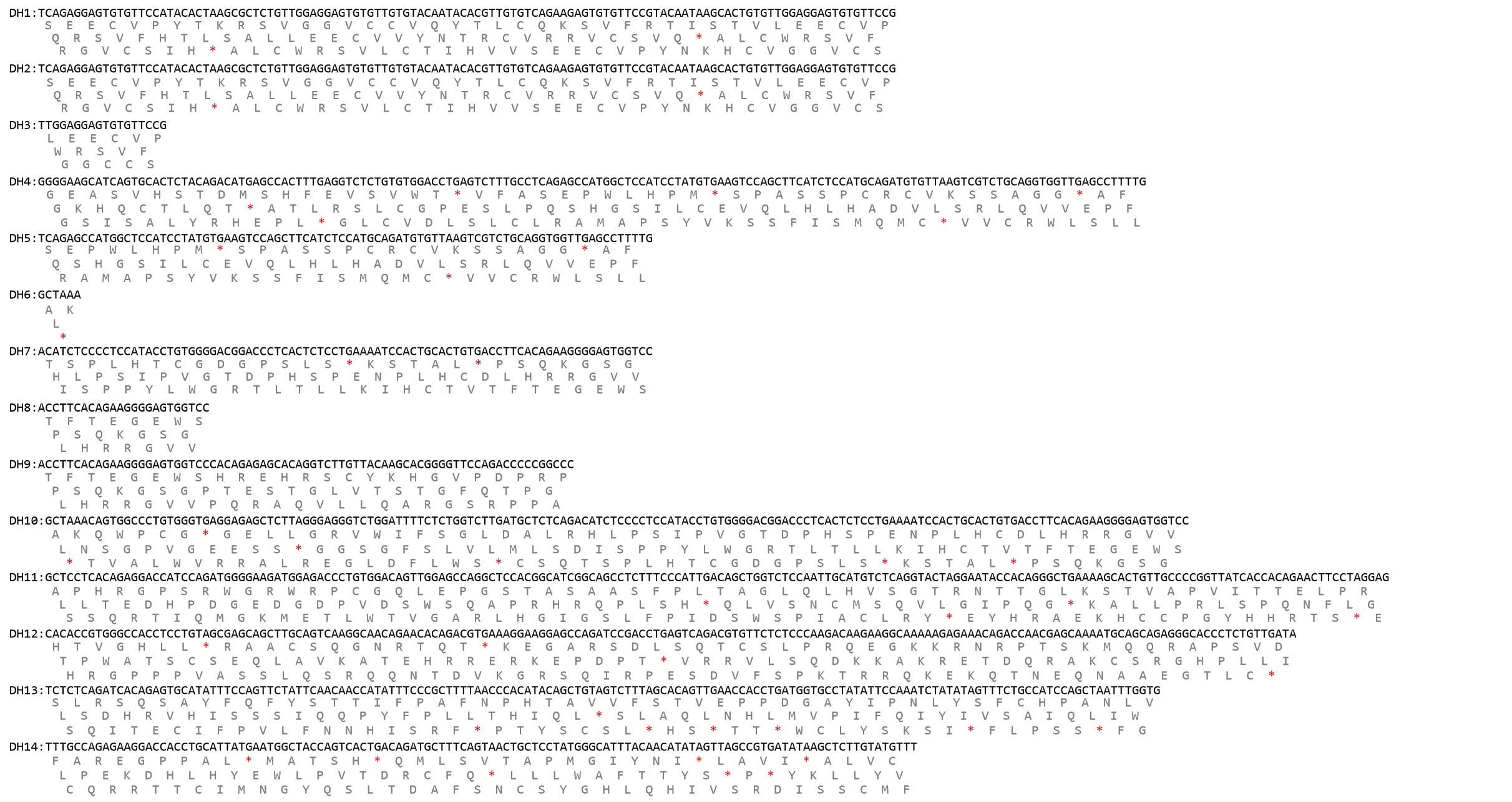

Supplement: Supplement 1 — DEGs list [file DataSheet_1.zip › Figure S1.jpg]

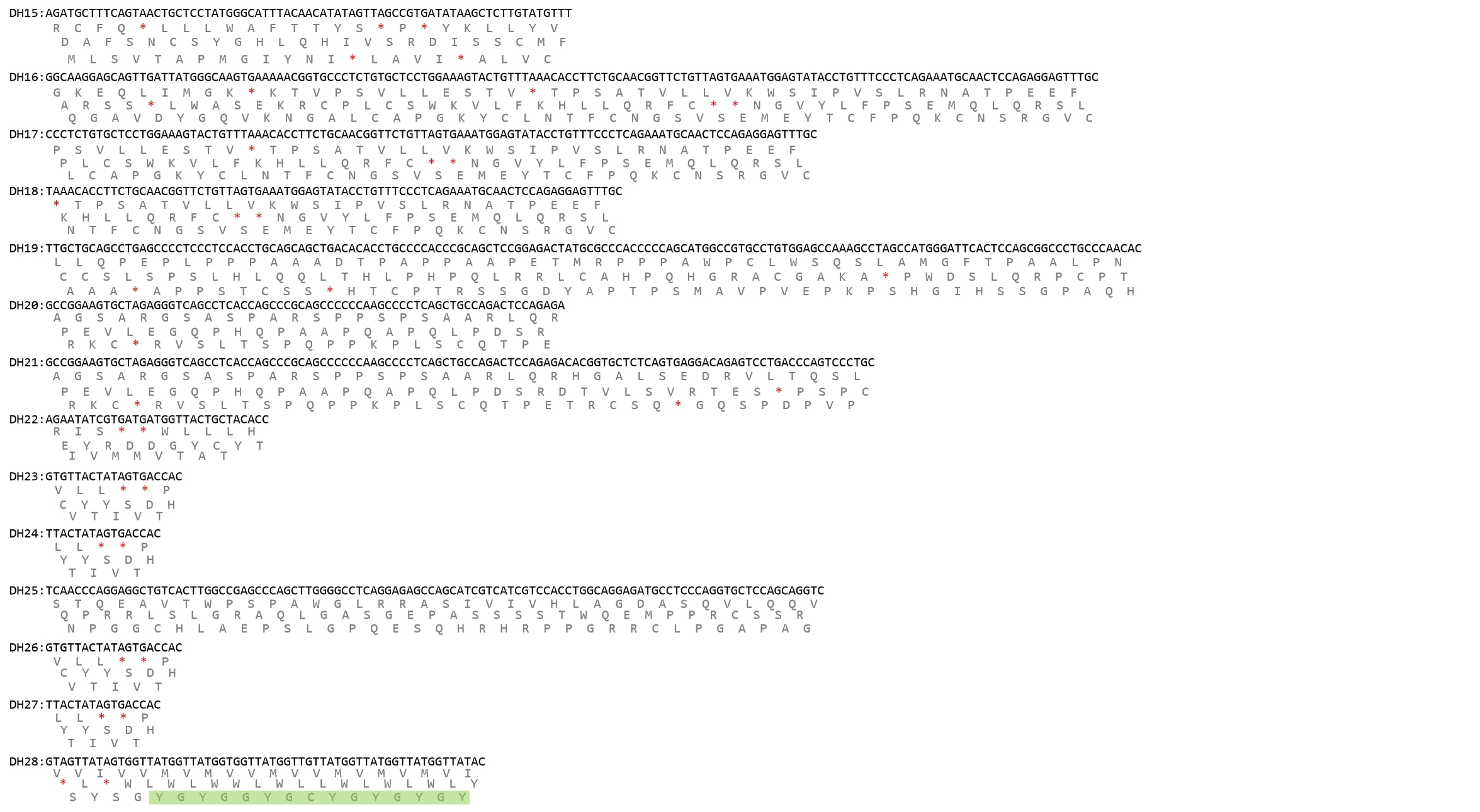

Supplement: Supplement 1 — DEGs list [file DataSheet_1.zip › Figure S2.jpg]

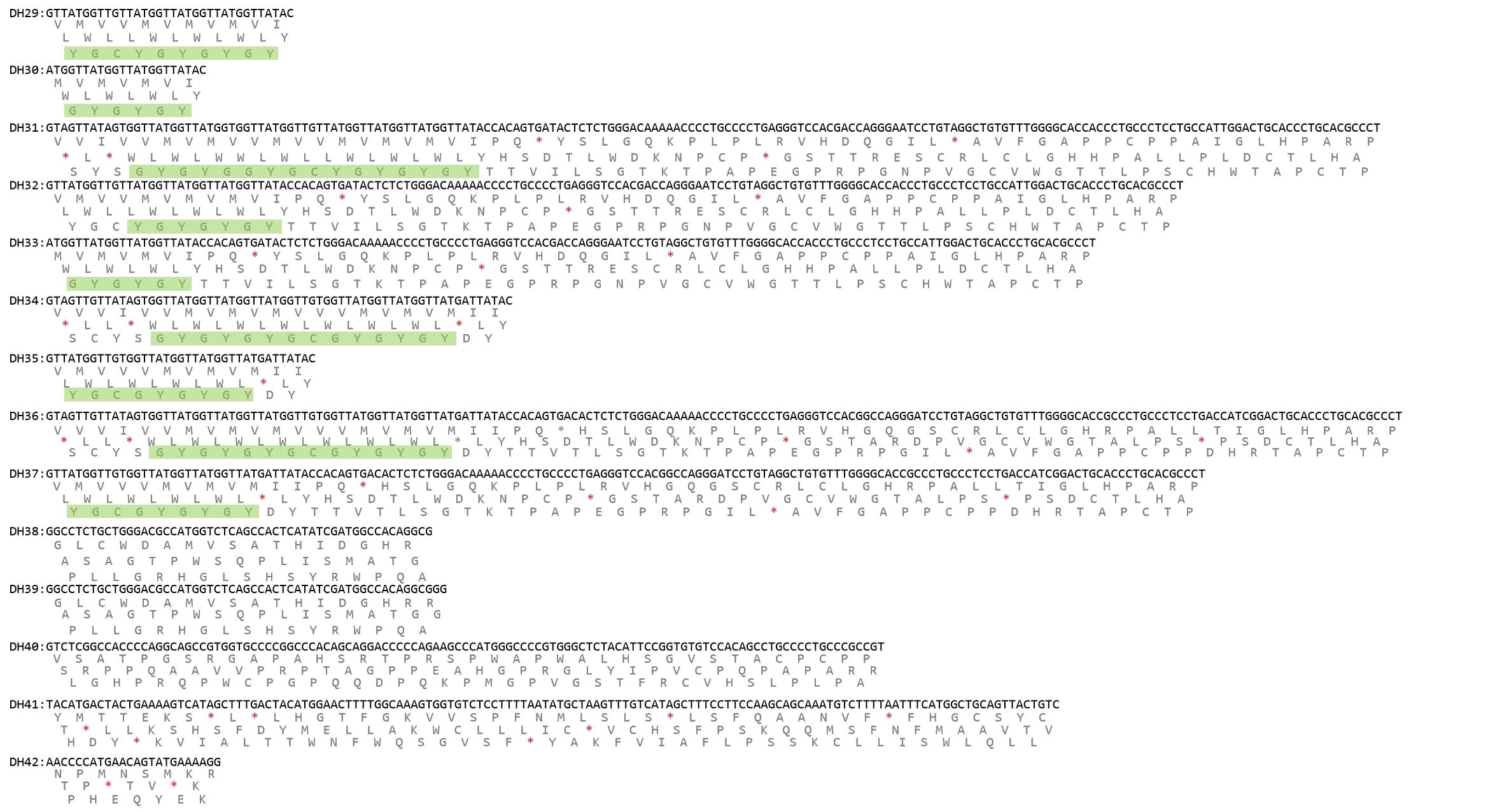

Supplement: Supplement 1 — DEGs list [file DataSheet_1.zip › Figure S3.jpg]

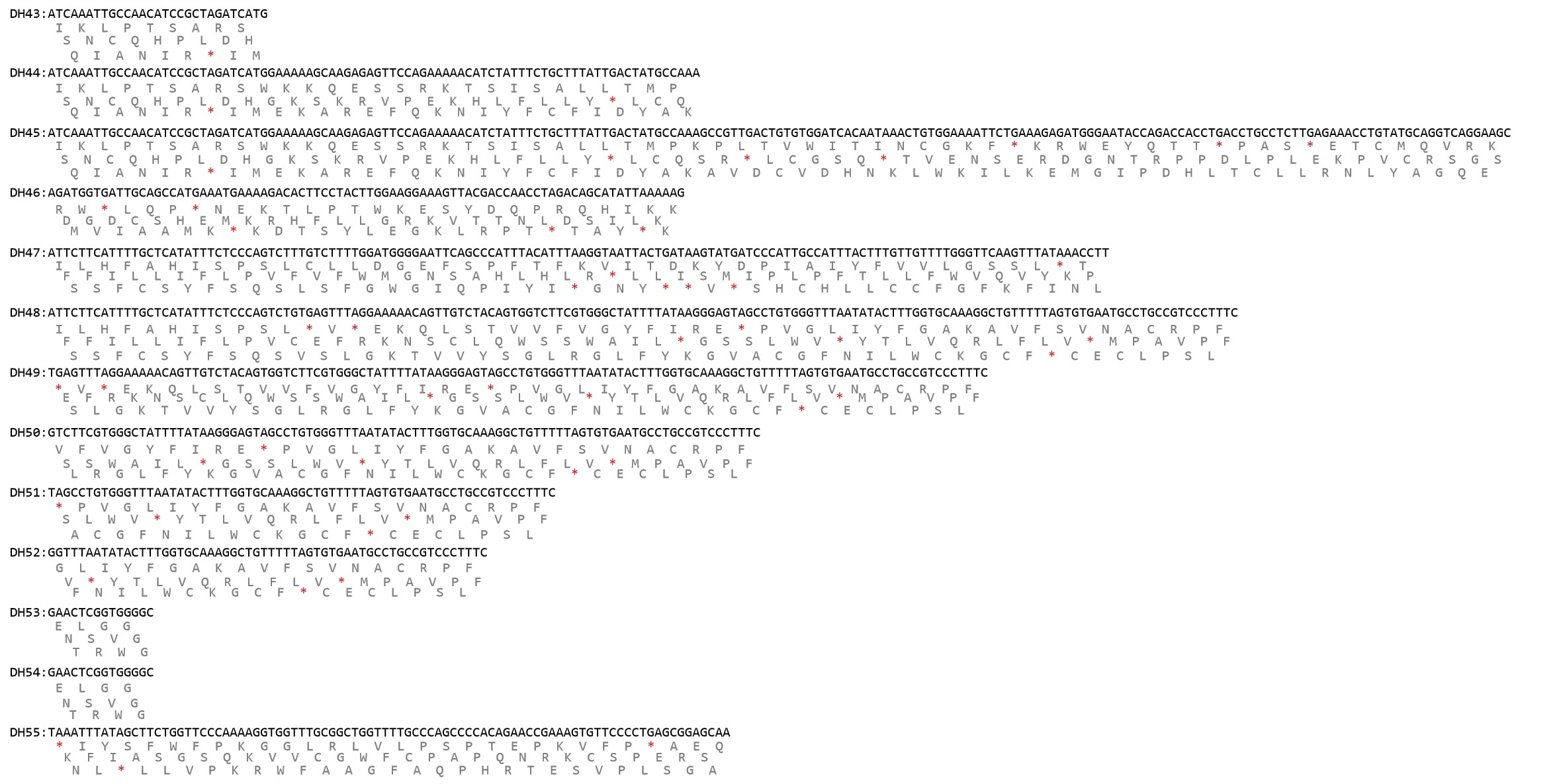

Supplement: Supplement 1 — DEGs list [file DataSheet_1.zip › Figure S4.jpg]
